# The evolution of self and hetero-evaluation of repeated high fidelity simulation scenarios of post-partum hemorrhage with anesthesia trainees

**DOI:** 10.15694/mep.2021.000160.1

**Published:** 2021-06-07

**Authors:** Emanuele Capogna, Denise Raccis, Francesco Salvi, Matteo Velardo, Giorgio Capogna

**Affiliations:** 1 EESOA Simulation Center

**Keywords:** Simulation, Medical Education

## Abstract

This article was migrated. The article was marked as recommended.

Introduction

Evidence for self-assessment in medicine is controversialwith participants under-rating or overestimating their performance. It is also unclear whether this under or overestimation changes during the process of repeated simulation experience. In this study, the authors analyzed the evolution of the behavioral skills of anesthesia trainees during four consecutive standardized postpartum hemorrhages (PPH) high-fidelity simulation scenarios. They compared the self-assessment made by the leader himself and the assessment made by his teammates individually with the assessment made by two expert observers.

Methods

The authors enrolled forty anesthesia trainee volunteers and divided them into eight teams of five participants each. Each team enacted the same scenario of a patient with atonic PPH following vaginal delivery four times so that all the trainees, except the one assigned the leader’s role, could rotate through the roles of anesthesia trainee, obstetrician, midwife, and nurse. The participants themselves and two expert observers, using standardized checklists and questionnaires, carried out an evaluation of the technical (diagnosis and treatment of atonic PPH) and behavioral (leadership, communication, situational awareness, and overall appraisal) skills evidenced in the scenarios.

Results

The authors noted a progressive improvement in the behavioral scores given to the leader by the examiners, his team, and himself, from the first to the fourth scenario. The scores given by the participants and by the leader himself were greater than those given by the independent observers in the first two scenarios but these differences were no longer significant during the last two scenarios.

Discussion

Participants overestimated their performances but this overestimation disappeared after the completion of the first two scenarios. The authors suggested that improving the skills of participants throughout the scenarios, most likely improved their metacognitive competence, helping them to better recognize their abilities.

## Introduction

Evaluation is a crucial matter in simulation. The quality of care eventually provided by learners depends on the quality of the teaching they have received and the learning experiences they have undergone, so an objective measure of the transfer of learning and an appropriate corrective activity are important components of the learning process.

In the second level of assessment of Kirkpatrick’s pyramid model ( Smidt *et al.*, 2019), instructors may evaluate the achievements directly related to the training by self or hetero-evaluation. The acquired skills may include knowledge, technical skills, and behavior (self-confidence, teamwork, leadership, CRM, etc).

Evidence for self-assessment in surgery is controversial ( Harrington, Murnaghanand and Regehr, 1997; Moorthy *et al.*, 2006) and studies in higher education have shown poor correlations between self and expert assessment with participants generally under-rating their performance ( Falkikov and Boud, 1989).

Conversely, another study has reported that trainees overestimate their ability when compared to the independent assessment ( Pandey *et al.*, 2008). However, it is not clear whether this under or overestimation changes during the process of repeated experience in simulation.

The primary aim of this study was to evaluate the evolution of the behavioral performance of the leader during four consecutive identical standardized postpartum hemorrhages (PPH) high-fidelity simulation scenarios. For the purpose of the study, we compared the self-assessment made by the leader himself and the assessment made by his teammates individually with the assessment made by two expert observers not involved in the scenarios.

## Methods

The authors registered the study at Clinical.Trial.Gov (ID n. NCT04398602).

Forty anesthesia trainees volunteered from the University of Catania Medical School to be enrolled in this prospective, observational study. Each participant gave written informed consent and privacy, confidentiality, and anonymity were fully guaranteed by the EESOA Research Board.

In our Region simulation centers do not have access to a formal ethical approval process. However, even if we did not have the ability to apply to an ethics committee for this work we have carried it on according to the Declaration of Helsinki. Our simulation center adheres and follows the Healthcare Simulationist Code of Ethics supported by the Society for Simulation in Healthcare ( Healthcare Simulationist Code of Ethics, 2018). Our study was eligible for the exemption, in accordance with US Federal Human Subject Regulations- Protection of Human Subjects, due to the nature of the study itself, as no patients were involved, the trainees participating were volunteers, researchers ensured that those taking part in the research would not be caused distress, all participants’ personal and other data were completely anonymized, and all the investigators had no conflict of interest and were not involved in any of the participants’ university teaching programs.

We studied eight teams each containing eight participants. The trainees enacted the same scenario of a patient with atonic PPH following vaginal delivery four times to enable all the trainees, except the one assigned the leader’s role (senior anesthesiologist’s role), to rotate through the roles of anesthesia trainee, obstetrician, midwife, and nurse.

The evaluation of the technical skills (diagnosis and treatment of atonic PPH) and behavioral, non-technical skills (leadership, communication, situational awareness, and overall appraisal) was made, in a double-blinded fashion, by the participants themselves and by two expert observers, not involved in the scenarios, who reviewed the video-recordings of the scenarios.

The technical skills of the team were evaluated on the basis of the completion of a PPH checklist. For the design of this checklist, we reviewed PPH guidelines from recognized obstetric bodies and literature, relevant papers from the literature, and their institutional PPH protocol ( Committee on Practice Bulletins-Obstetrics, 2017; Mavrides *et al.*, 2016; Queensland Clinical Guidelines, 2019; Hilton *et al.,* 2016).

**Figure 4:**
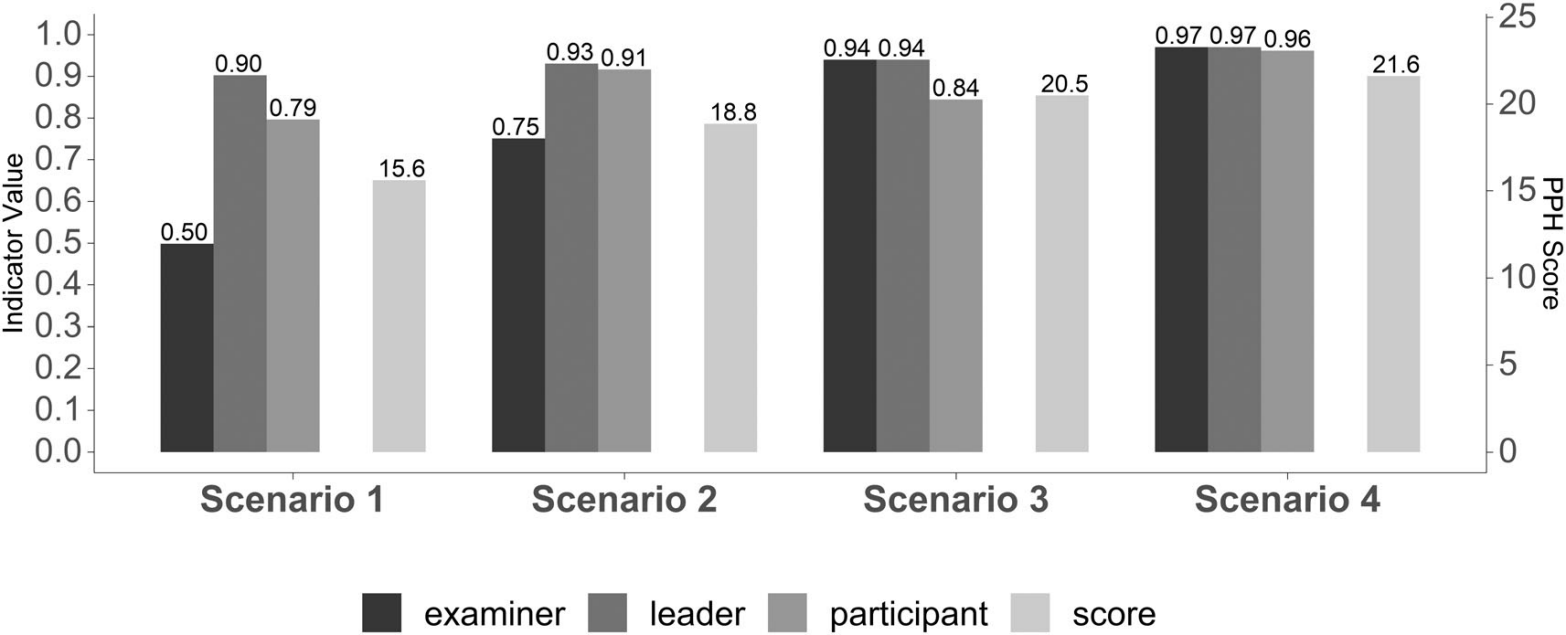
Overall team performance (left scale) and the PPH scores (right scale). Answer Agreement Indicator (AI). (Statistical significance in Table 1).

We then chose, by consensus, the final action items for the checklist, identifying 25 standardized key tasks for inclusion on the PPH checklist (technical skills). This checklist worked as the reference guide for pre-scenario briefing and for the team’s technical skills evaluation during the scenario (Appendix 1). We also developed standardized questionnaires for the evaluation of behavioral skills. (Appendix 2-4). For the design of these evaluation and self-evaluation questionnaires, we reviewed the non-technical skills evaluation instruments, such as the Anaesthetists’ Non-Technical Skills (ANTS) behavioral marker system and the Ottawa Global Rating Scale (GRS) ( Jirativanont *et al.*, 2017).

We then selected, by consensus, the final items for the questionnaire.

No formal training took place before the first scenario, in order to consider the first scenario as the participants’ baseline performance. All the teams underwent standardized educational training immediately before the second, third, and fourth scenarios. Every standardized educational training consisted of a 30-minute session. At the beginning of each session, each participant was invited to write on a piece of paper, in three minutes, what they would do in the case of postpartum bleeding. They were then invited to share and to write on the blackboard, one by one, in turn, the clinical procedures that they had previously reported in their personal papers. After this session, the instructor, after having asked all the participants whether there was something else to add, discussed and integrated, if necessary, the missing elements by using the 25 key tasks (Appendix 1) in order to have a shared check-list of the postpartum hemorrhage treatment to be practiced during the scenario. All the teams received a standardized lesson on crisis resource management (CRM) before the second scenario ( Howard *et al.*, 1992).

The scenario consisted of a severe PPH (>1500 mL blood loss) due to refractory uterine atony in a multiparous 28-year-old patient who had undergone a spontaneous vaginal delivery. The patient became tachycardic and hypotensive consistent with hemorrhagic shock. All simulations were performed in the simulation room of the EESOA Simulation Center (Rome) using a high-fidelity manikin (Sim Mom Maternal and Neonatal Birthing Simulator, Laerdal, Norway). All simulations were videotaped. The simulation was stopped when each team had completed all 25 tasks of the checklist, or when 15 minutes had elapsed. Each scenario was followed by a standardized debriefing led by an expert debriefer.

A study investigator, an expert in both PPH and simulation debriefing and not involved in the simulation, observed each scenario in the control room, to record and check the team’s technical skills (PPH evaluation and treatment), according to the established 25 PPH key tasks (Appendix 1). The behavioral scores (Appendix 2) were assigned by two observers, experts in communication and evaluation in simulation, and not involved in the scenarios, who reviewed the videos of each simulation. After each simulation, all participants were asked to complete a survey concerning the leader and the team behavior, and to the overall performance. Questions required a yes/no answer or an answer based on an ordinal scale (Appendix 3 and 4).

### Statistical Analysis

To compare the frequency distribution of the questionnaire items, we created an answer agreement indicator (AI) ( Agresti, 1984; Aiello and Attanasio, 2004; Saisana and Tarantola, 2002) considering a transformation of the original data, which respected their ordinal nature and the variability of the distributions. The indicator was based on the distance between an observed distribution and theoretical distribution under optimal conditions, i.e. maximum concordance for the best judgment:

**Figure:**
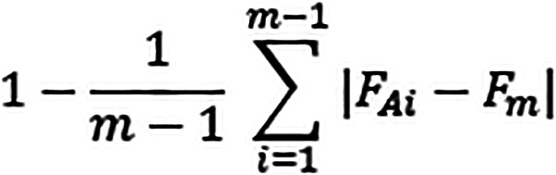


m = number of modes of the variable; FAi = observed distribution; Fm = theoretical distribution.

This AI is sensitive to the values assumed by the dispersion of the different distributions and the average level:

**Figure:**
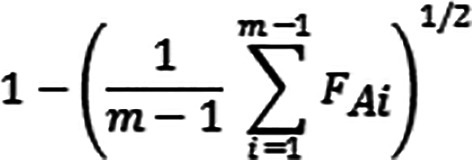


The AI standardizes in a single measure the average and the variability of the given as a result value: 0≤AI≤1, where 0 indicates maximum concordance towards the worst judgment, and 1 indicates the maximum concordance towards the best judgment. The AI was also used for the generation of rankings, so enabling us to correlate leader, participant, and examiner evaluations between scenarios by using the correlation coefficient for Spearman’s Rho rank. We used the Mann Whitney U-test to examine the differences between the individual scores in each scenario.

## Results/Analysis

All the participants successfully completed the scenarios and answered the survey.

**Figure 1:**
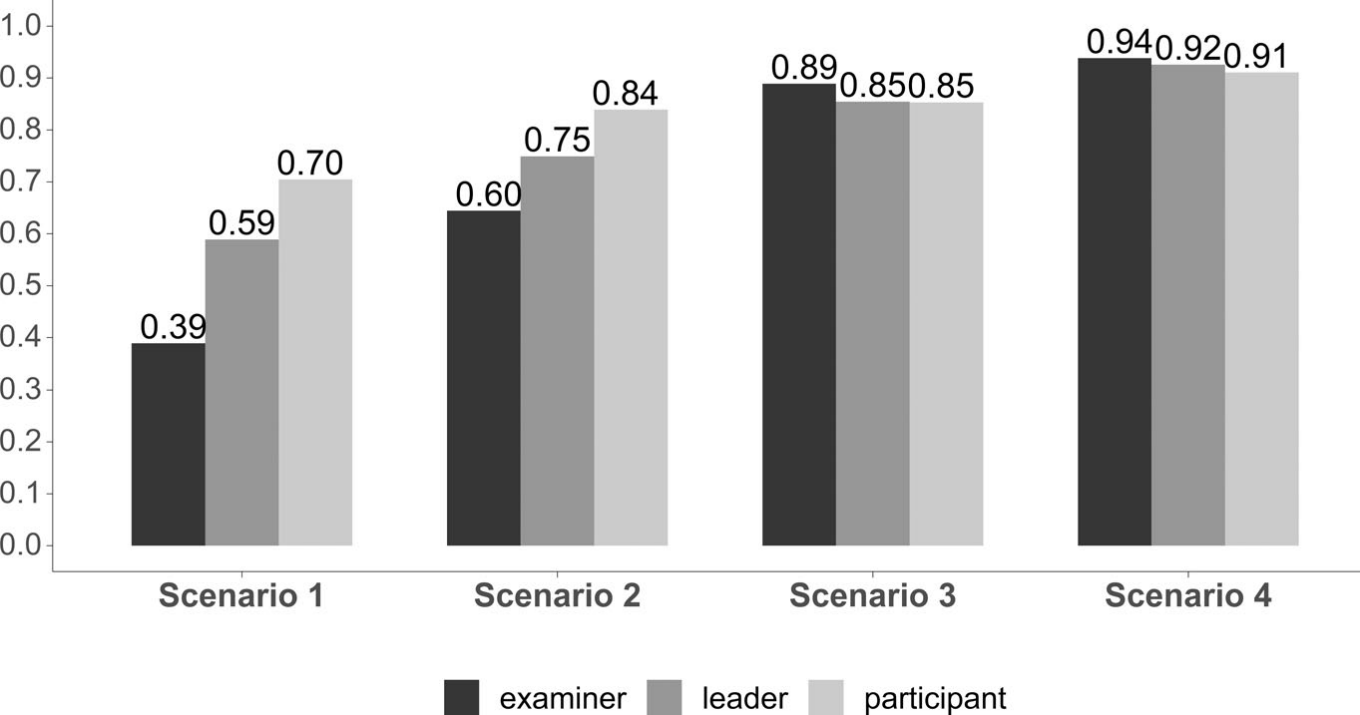
Leadership skills. Answer Agreement Indicator (AI). (Statistical significance in Table 1).

**Figure 2:**
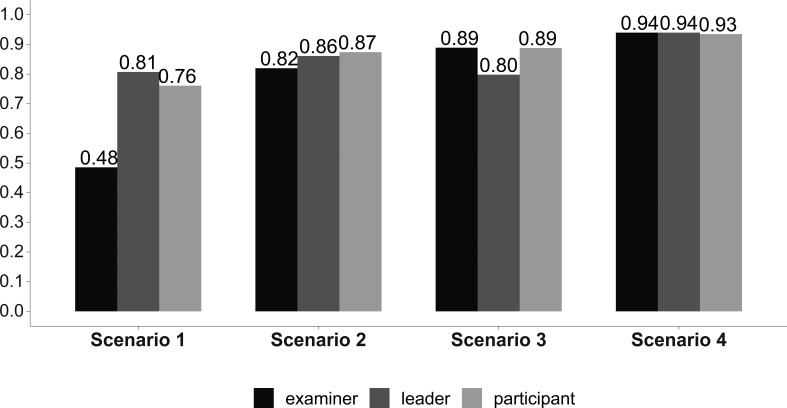
Situational awareness skills. Answer Agreement Indicator (AI). (Statistical significance in Table 1).

**Figure 3:**
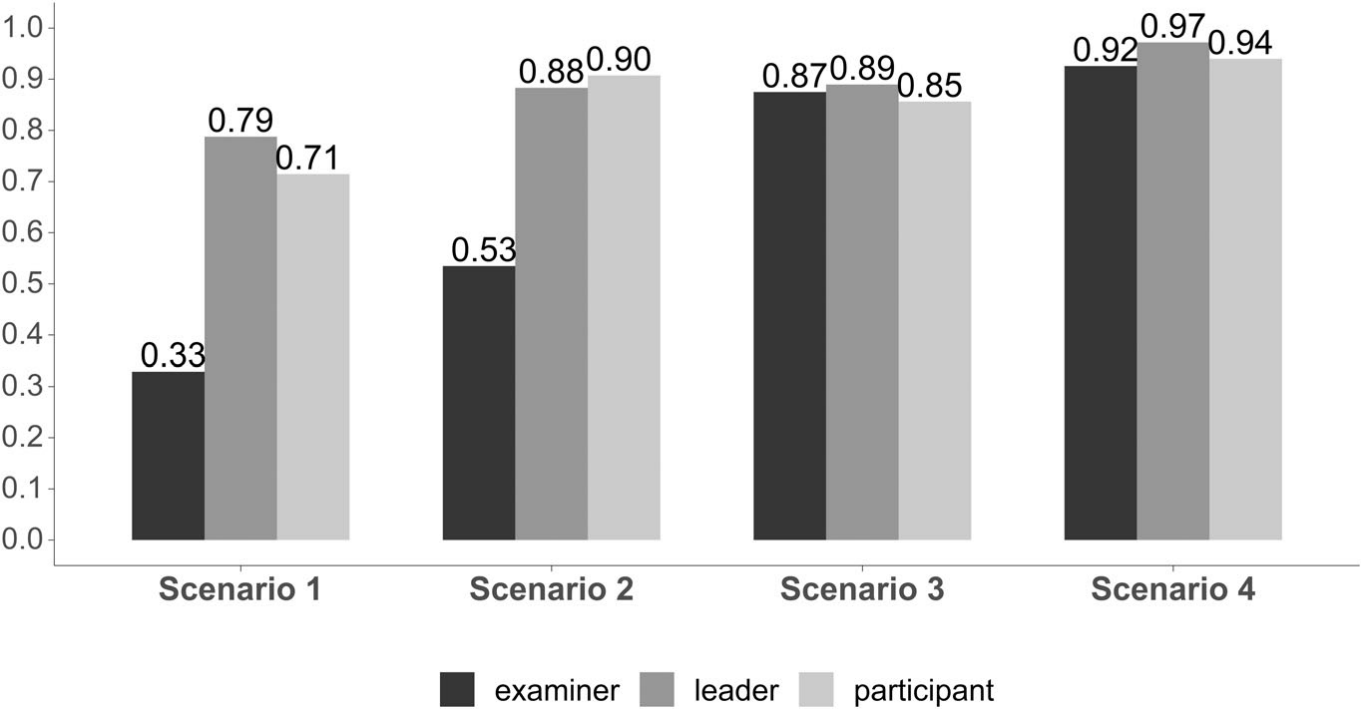
Communication skills. Answer Agreement Indicator (AI.) (Statistical significance in Table 1).

In Figures 1- 4 we reported the indicator (AI) values of the leader’s self-evaluation, the participants’ evaluation of the leader, and of the expert examiners evaluations after the four scenarios, concerning leadership ( Figure 1), situational awareness ( Figure 2), communication skills ( Figure 3) and overall team performance and PPH scores ( Figure 4). Statistical significance of the values of the leader’s self-evaluation, the participants’ evaluation of the leader and of the expert examiners’ evaluations during the four scenarios, concerning leadership, situational awareness, communication skills and overall team performance is reported in Table 1.

**Table 1:** Statistical significance of the values of the leader’s self-evaluation, the participants’ evaluation of the leader and of the expert examiners’ evaluations during the four scenarios.

Participant Evaluation (PE) - Examiner Evaluation (EE)					
Skills and Performance	Rho	Scenario 1	Scenario 2	Scenario 3	Scenario 4
Leadership	1,0 (P=.03)	PE > EE (P<.001)	PE > EE (P=.01)	PE ≠ EE (P=.88)	PE ≠ EE (P=.94)
Awareness	1,0 (P=.01)	PE > EE (P<.001)	PE ≠ EE (P=.15)	PE ≠ EE (P=.70)	PE ≠ EE (P=.87)
Communication	0,8 (P=.06)	PE > EE (P<.001)	PE > EE (P<.001)	PE ≠ EE (P=.22)	PE ≠ EE (P=.28)
Team Performance	0,8 (P=.03)	PE >EE (P=.03)	PE > EE (P=.05)	PE < EE (P<.001)	PE ≠ EE (P<.001)

Leader Evaluation (LE) - Examiner Evaluation (EE)					
Skills and Performance	Rho	Scenario 1	Scenario 2	Scenario 3	Scenario 4
Leadership	1,0 (P<.001)	LE > EE (P=.01)	LE > EE (P=.05)	LE ≠ EE (P=.88)	LE ≠ EE (P=.99)
Awareness	0,4 (P=.02)	LE > EE (P<.001)	LE ≠ EE (P=.22)	LE ≠ EE (P=.90)	LE ≠ EE (P=.50)
Communication	1,0 (P=.06)	LE > EE (P<.001)	LE > EE (P<.001)	LE ≠ EE (P=.45)	LE ≠ EE (P=.16)
Équipe Performance	1,0 (P=.03)	PE > EE (P=.04)	LE > EE (P=.05)	LE ≠ EE (P=.70)	LE ≠ EE (P=.58)
Leader Performance	1,0 (P=.04)	LE > EE (P=.03)	LE > EE (P=.72)	LE ≠ EE (P=.40)	LE ≠ EE (P=.32)

Participant Evaluation (PE) - Leader Evaluation (LE)					
Skills and Performance	Rho	Scenario 1	Scenario 2	Scenario 3	Scenario 4
Leadership	1,0 (P<.001)	PE > LE (P=.07)	PE > LE (P=.04)	PE ≠ LE (P=.60)	PE ≠ LE (P=.17)
Awareness	0,4 (P<.001)	PE ≠ LE (P=.66)	PE ≠ LE (P=.96)	PE ≠ LE (P=.28)	PE ≠ LE (P=.26)
Communication	0,8 (P<.001)	PE ≠ LE (P=.70)	PE ≠ LE (P=.45)	PE ≠ LE (P=.53)	PE ≠ LE (P=.50)
Team Performance	0,8 (P<.001)	PE < LE (P=.41)	PE ≠ LE (P=.49)	PE ≠ LE (P=.30)	PE ≠ LE (P=.63)

**Figure 5:**
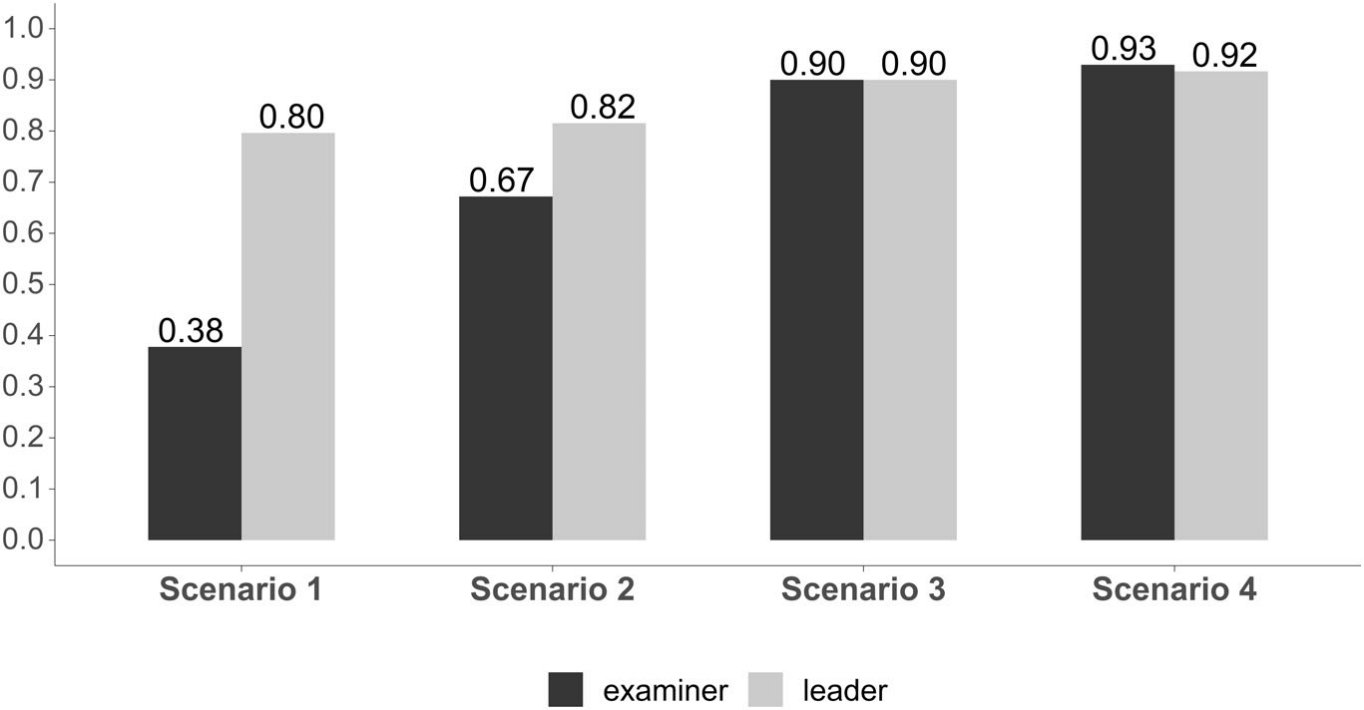
Leader’s overall performance. Answer Agreement Indicator (AI). (Statistical significance in Table 1).

Correlations between leader, participants and examiners’ evaluations between scenarios were examined by coefficient for Spearman’s Rho rank. Differences between the individual scores in each scenario were examined by the Mann Whitney U-test. Indicators and data values are reported in the Figures 1- 5.

We noted significant progressive improvement in the scores given to the leader by the independent examiners, by his team, and by himself from the first to the fourth scenario, indicating a consensus in leader performance improvement as he completed the scenarios ( Table 1). The scores given by the participants and by the leader himself were greater than those given by the independent observers in the first two scenarios ( Table 1). These differences were no longer significant during the last two scenarios ( Table 1). The overall team performance improved from the first to the fourth scenario ( Table 1 and Figure 4). PPH checklist scores improved from the first to the fourth scenario ( Figure 4), according to the leader’s score (Rho = 1, P<.01), participant’s score (Rho = 0.8, P<.01), and examiner’s score (Rho = 1, P<.01).

The overall leader’s performance improved from the first to the fourth scenario but it was overestimated in the first two scenarios ( Table 1 and Figure 5).

## Discussion

In this study, the performance of trainees (behavioral assessment) who had enacted the role of a leader in a simulation scenario was evaluated by themselves, by their peer trainees who had been in their team in the scenario, and by two external expert assessors who reviewed the videos of the simulations. This study design gave us the chance to compare, using the same questionnaire, the self, the peer, and expert evaluation. In addition, the evaluation was carried out four times in a row after four identical scenarios preceded by briefings and followed by standardized debriefings, allowing us to observe the time evolution of the given scores as the trainees gained experience through progressive training. As the team underwent a technical assessment (by using the PPH checklist), we were also able to observe the evolution of the technical performance of each leader’s team.

As expected, the overall leader’s performance and the technical performance of the team improved from the first to the fourth scenario, and this improvement was consistently recorded by the leader himself, by his teammates, and by the observers.

However, this improvement was overestimated in the leader’s self-assessment when compared with the observers’ indicator scores. Interestingly such an overestimation was also noted in the teammate’s assessments, but in both cases, this overestimation was present only in the first two scenarios.

It is commonly believed that adult learners are aware of what they know and how well they perform. However, studies of self -assessment of professional performance, observed on videotape and rated behaviorally, were associated with moderately high validity and, under certain conditions, with emotionally gratifying self-identification of needed improvements ( Gordon, 1991).

Despite its common use, people’s self-perceptions often deviate from how others perceive them and how they actually behave. Research on self-assessment has shown that individuals’ appraisals of their ability in a given domain often bear little relationship to how they perform on tests of that ability or on others’ assessments of their ability ( Carter and Dunning, 2008) leading to low accuracy of self-assessment ( Sitzman *et al.*, 2010).

In one study by Moorthy *et al.*(2006) surgical trainees performed a simulated surgery on a synthetic model in a simulated operating theater. There was a strong correlation between the experts’ rating of technical skills and the self-assessment of the participants, and this correlation improved with increasing experience. However, there was a low correlation between the self-assessment and the experts’ scores for human factors skills.

Risucci, Tortolani, and Ward (1989) compared self, peer, and supervisor ratings with scores on the American Board of Surgery In-Training Examination (ABSITE), and self and peer ratings were lower than ratings by the supervisor.

We have observed that the difference between the self and ethero-assessment was attenuated by the experience gained until there was no difference in the last scenarios.

It has been reported that unskilled people may have metacognitive deficits such as the overall tendency towards overestimated self-appraisals ( Kruger and Dunning, 1999). As a result, they will tend to grossly overestimate their skills and abilities. Whether the task is difficult because of its nature or because the person is unskilled, the end result is a large degree of overconfidence.

In their first scenario, our trainees were in a similar condition, and this may have contributed to their initial overestimation of their behavioral skills.

This may confirm the hypothesis that self-evaluation may be regarded as a skill and, as such, needs time to be developed ( Fuhrmann and Weissburg, 1978). Improving the skills of participants throughout the scenarios, most likely improved their metacognitive competence, helping them to better recognize their abilities ( Kruger and Dunning, 1999).

Our findings suggest that the studies based only on a single self-evaluation by medical trainees should probably be interpreted cautiously.

Nevertheless, it should be remembered that self-assessment can be a valuable learning activity in itself, even in the absence of significant agreement between trainee and expert observer, and can provide potent feedback to the trainee about both learning and educational and professional standards.

## Conclusion

In conclusion, we reported progressive improvement in the scores given to the leader both by the independent examiners, by his team, and by himself from the first to the fourth scenario. Participants overestimated their performances but this overestimation disappeared after the completion of the first two scenarios. We believe these findings may contribute to the planning of protocols to prepare learners for self-regulated learning in simulation and clinical setting training.

## Take Home Messages

• Participants overestimated their performances but this overestimation disappeared after the completion of the first two scenarios.
• Improving the skills of participants throughout the scenarios, most likely improved their metacognitive competence, helping them to better recognize their abilities.
• These findings may contribute to the planning of protocols to prepare learners for self-regulated learning in simulation and clinical setting training.

## Notes On Contributors

**Emanuele Capogna** is a Debriefer and Simulation Specialist at EESOA Simulation Center, Roma, Italy. ORCiD: https://orcid.org/0000-0002-4053-1274

**Denise Raccis,** PhD is a Psychologist and Communication Specialist at EESOA Simulation Center, Roma, Italy. ORCiD: https://orcid.org/0000-0001-5341-3544

**Francesco Salvi,** PhD is a Simulation Technician, EESOA Simulation Center, Rome, Italy. ORCiD: https://orcid.org/0000-0002-6129-4482

**Matteo Velardo,** PhD is a Statistician, EESOA Simulation Center, Rome, Italy. ORCiD: https://orcid.org/0000-0001-9832-6276

**Giorgio Capogna,** MD is Director of the European School of Obstetric Anesthesia and of EESOA, Maternal Neonatal Simulation Center, Rome, Italy. ORCiD: https://orcid.org/0000-0003-2298-8905

## Appendices

### Appendix 1: Atonic PPH Check List

◻ Call for Help
◻ IV - place 2nd large bore
◻ Stat Labs CBC + coagulation + TEG
◻ Consider OR
◻ Bakri Balloon
◻ Vasopressors
◻ Fundal massage
◻ Bimanual compression
◻ Oxytocin
◻ Fluids
◻ Oxygen
◻ Vital Signs Assessment
◻ 4 T Assessment
◻ Transfusions: Blood, Fresh Frozen Plasma, Cryoprecipitates
◻ Place Foley
◻ I Line Uterotonics
◻ II Line Uterotonics
◻ Tranexamic acid
◻ Fibrinogen
◻ Blood gas analysis
◻ Blood loss evaluation
◻ Warming
◻ Inform patient & relatives
◻ ABCD
◻ Consider Intensive Care Unit/ Interventional Radiology

### Appendix 2: Examiners’ Assessment

#### Leadership

Did the Leader Anesthesiologist coordinate everyone’s work?

4=always; 3=often; 2= sometimes; 1=never

Did the Leader Anesthesiologist set priorities?

4=always; 3=often; 2= sometimes; 1=never

Did the Leader Anesthesiologist distribute the workloads to the team members?

4=always; 3=often; 2= sometimes; 1=never

#### Situational Awareness

Did the Leader Anesthesiologist anticipate the clinical problems?

4=always; 3=often; 2= sometimes; 1=never

Did the Leader Anesthesiologist listen to all the suggestions given by the members of the group?

4=always; 3=often; 2= sometimes; 1=never

Did the Leader Anesthesiologist reassess the patient’s condition several times?

4=always; 3=often; 2= sometimes; 1=never

#### Communication

Did the Leader Anesthesiologist share his intervention strategy, in terms of diagnosis and therapy, with the rest of the team?

4=always; 3=often; 2= sometimes; 1=never

Did the Leader Anesthesiologist communicate succinctly and clearly?

4=always; 3=often; 2= sometimes; 1=never

Did the Leader Anesthesiologist look you in the eye when you spoke to him and/or did you call him by his name?

4=always; 3=often; 2= sometimes; 1=never

#### Overall Assessment

In general, the performance of the Leader Anesthetist was:

5=excellent; 4=good; 3= sufficient; 2=insufficient; 1= poor

In general, the performance of the team was:

5=excellent; 4=good; 3= sufficient; 2=insufficient; 1=poor

EPP Checklist score (1-25)

### Appendix 3: Leader (Senior Anesthesiologist) Self-Assessment

#### Leadership

Did you coordinate everyone’s work?

4=always; 3=often; 2= sometimes; 1=never

Did you prioritize?

4=always; 3=often; 2= sometimes; 1=never

Did you distribute the workloads to the team members?

4=always; 3=often; 2= sometimes; 1=never

#### Situational Awareness

Did you anticipate the clinical problems?

4=always; 3=often; 2= sometimes; 1=never

Did you listen to all the suggestions given by the members of the group?

4=always; 3=often; 2= sometimes; 1=never

Did you reassess the patient’s condition several times?

4=always; 3=often; 2= sometimes; 1=never

#### Communication

Did you share his intervention strategy, in terms of diagnosis and treatment, with the rest of the team?

4=always; 3=often; 2= sometimes; 1=never

Did you communicate with the team in a concise and clear manner?

4=always; 3=often; 2= sometimes; 1=never

Did you look them in the eye when you communicated with them and/or called them by name?

4=always; 3=often; 2= sometimes; 1=never

Did you verify that the team members had complied with your requests?

4=always; 3=often; 2= sometimes; 1=never

#### Overall Assessment

Your performance was: 4=excellent; 3=good; 2=insufficient;1=poor

In general, the performance of the team was: 4=excellent; 3=good; 2=insufficient; 1=poor

### Appendix 4: Participant’s Assessment of the Leader

#### Leadership

Did the Leader Anesthesiologist coordinate everyone’s work?

4=always; 3 Leader =often; 2= sometimes; 1=never

Did the Leader Anesthesiologist set priorities?

4=always; 3=often; 2= sometimes; 1=never

Did the Leader Anesthesiologist distribute the workloads to the team members?

4=always; 3=often; 2= sometimes; 1=never

#### Situational Awareness

Did the Leader Anesthesiologist anticipate clinical problems?

4=always; 3=often; 2= sometimes; 1=never

Did the Leader Anesthesiologist listen to all the suggestions given by the team members?

4=always; 3=often; 2= sometimes; 1=never

Did the Leader Anesthesiologist reassess the patient’s condition several times?

4=always; 3=often; 2= sometimes; 1=never

#### Communication

Did the Leader Anesthesiologist share her intervention strategy, in terms of diagnosis and therapy, with the rest of the team?

4=always; 3=often; 2= sometimes; 1=never

Did the Leader Anesthesiologist communicate in a concise and clear way?

4=always; 3=often; 2= sometimes; 1=never

Did the Leader Anesthesiologist have eye contact with the person talking to him and/or did he call him by name?

4=always; 3=often; 2= sometimes; 1=never

#### Overall Assessment

In general, the team’s performance was: 4=excellent; 3=good; 2=insufficient; 1=poor

## References

[ref1] Aiello F. Attanasio M. (2004) How to transform a batch of simple indicators to make up a unique one? Atti del Convegno SIS, Bari, Sessioni Specializzate.pp.327–338.

[ref2] Agresti A. (1984) Analysis of ordinal categorical data. New York: Wiley.

[ref3] Carter T. J. and Dunning D. (2008) Faulty self-assessment: Why evaluating one’s own competence is an intrinsically difficult task. Social and Personality Psychology Compass. 2(1), pp.346–360. https://doi.org/10.1111/j.1751-9004.2007.00031.x

[ref4] Committee on Practice Bulletins-Obstetrics (2017). Practice Bulletin No. 183: Postpartum Hemorrhage. Obstetrics and Gynecology. 130(4),e168–e186. https://doi.org/10.1097/AOG.0000000000002351 28937571 10.1097/AOG.0000000000002351

[ref5] Falchikov N. and Boud D. (1989) Student Self-Assessment in Higher Education: A Meta-Analysis. Review of Educational Research. 59(4), pp.395–430. https://doi.org/10.3102/00346543059004395

[ref6] Fuhrmann B. and Weissburg M. (1978) Self-evaluation.In: Morgan M. and Irby D. eds. Evaluating Clinical Competence in the Health Professions. St Louis, Missouri: Mosby.

[ref7] Gordon M. J. (1991) A review of the validity and accuracy of self-assessments in health professions training. Academic Medicine: Journal of the Association of American Medical Colleges. 66(12), pp.762–769. https://doi.org/10.1097/00001888-199112000-00012 1750956 10.1097/00001888-199112000-00012

[ref8] Harrington J. P. Murnaghan J. J. and Regehr G. (1997) Applying a relative ranking model to the self-assessment of extended performances. Advances in Health Sciences Education: Theory and Practice. 2(1), pp.17–25. https://doi.org/10.1023/A:1009782022956 16180055 10.1023/A:1009782022956

[ref9] Healthcare Simulationist Code of Ethics . (2018) Society for Simulation in Healthcare Guidelines. Available at: https://www.ssih.org/SSH-Resources/Code-of-Ethics( Accessed: 20 February 2021).

[ref10] Hilton G. Daniels K. Goldhaber-Fiebert S. N. Lipman S. (2016) Checklists and multidisciplinary team performance during simulated obstetric hemorrhage. International Journal of Obstetric Anesthesia. 25, pp.9–16. https://doi.org/10.1016/j.ijoa.2015.08.011 26421705 10.1016/j.ijoa.2015.08.011 PMC4727983

[ref11] Howard S. K. Gaba D. M. Fish K. J. Yang G. (1992) Anesthesia crisis resource management training: teaching anesthesiologists to handle critical incidents. Aviation, Space, and Environmental Medicine. 63(9), pp.763–770. 1524531

[ref12] Jirativanont T. Raksamani K. Aroonpruksakul N. Apidechakul P. (2017) Validity evidence of non-technical skills assessment instruments in simulated anaesthesia crisis management. Anaesthesia and Intensive Care. 45(4), pp.469–475. https://doi.org/10.1177/0310057X1704500410 28673217 10.1177/0310057X1704500410

[ref13] Kruger J. and Dunning D. (1999) Unskilled and unaware of it: how difficulties in recognizing one’s own incompetence lead to inflated self-assessments. Journal of Personality and Social Psychology. 77(6), pp.1121–1134. https://doi.org/10.1037//0022-3514.77.6.1121 10626367 10.1037//0022-3514.77.6.1121

[ref14] Mavrides E. Allard S. Chandraharan E. Collins P. (2016) on behalf of the Royal College of Obstetricians and Gynaecologists. ‘Prevention and management of postpartum haemorrhage’. British Journal of Obstetrics and Gynaecology. 124:e106–e149.27981719

[ref15] Moorthy K. Munz Y. Adams S. Pandey V. (2006) Self-assessment of performance among surgical trainees during simulated procedures in a simulated operating theater. American Journal of Surgery. 192(1), pp.114–118. https://doi.org/10.1016/j.amjsurg.2005.09.017 16769287 10.1016/j.amjsurg.2005.09.017

[ref16] Pandey V. A. Wolfe J. H. Black S. A. Cairols M. (2008) Self-assessment of technical skill in surgery: the need for expert feedback. Annals of the Royal College of Surgeons of England. 90(4), pp.286–290. 10.1308/003588408X286008 18492390 PMC2647188

[ref17] Smidt A. Balandin S. Sigafoos J. , & Reed V. A. (2009) The Kirkpatrick model: A useful tool for evaluating training outcomes. Journal of Intellectual & Developmental Disability. 34(3),266–274. https://doi.org/10.1080/13668250903093125 19681007 10.1080/13668250903093125

[ref18] Queensland Clinical Guidelines (2019), Primary Postpartum Haemorrhage. MN18.1-V8-R23. Available at: https://www.health.qld.gov.au/__data/assets/pdf_file/0015/140136/g-pph.pdf( Accessed: 20 February 2021).

[ref19] Risucci D. A. Tortolani A. J. and Ward R. J. (1989) Ratings of surgical residents by self, supervisors and peers. Surgery, Gynecology & Obstetrics. 169(6), pp.519–526. 2814768

[ref20] Saisana M. and Tarantola S. (2002) State-of-the-art report on current methodologies and practices for composite indicator development. Report EUR 20408 EN. European Commission-Joint Research Centre, ISPRA.Available at: https://www.eea.europa.eu/data-and-maps/indicators/economic-water-productivity-of-irrigated/state-of-the-art-report( Accessed: 20 February 2021).

[ref21] Sitzmann T. Ely K. Brown K. G. and Bauer K. (2010) Self-assessment of knowledge: A cognitive learning or affective measure? Academy of Management Learning & Education. 9(2), pp.169–191.

