# Supplementary material for: The evolution of self and hetero-evaluation of repeated high fidelity simulation scenarios of post-partum hemorrhage with anesthesia trainees
Source: MedEdPublish (2016). 2021 Jun 7;10:160. [Version 1] doi: 10.15694/mep.2021.000160.1 (PMC10939620; doi:10.15694/mep.2021.000160.1)
Supplement: Peer review history

# Reviewer report: Reviewer response for version 1

**DOI:** 10.21956/mep.18892.r26979

**Published:** 2021-08-03

**Authors:** Julie Hunt

**Affiliations:** 1 Lincoln Memorial University

This review has been migrated. The reviewer awarded 3 stars out of 5 This paper investigates the relationship between self-assessment, peer assessment, and expert assessment over a series of four identical runs of a single PPH simulation. Self-assessment by trainees is commonly known to be inaccurate, so this paper is interesting in its investigation of when in the simulation series the self-assessment became accurate. As such, it has relevance to all medical education simulations, although the point at which the self-assessment becomes accurate will no doubt vary based on the difficulty of the scenario for the level of trainee, and what type of debriefing is done.I did wonder why self-assessment in surgery was highlighted in the introduction and discussion. Perhaps there is not relevant literature in anesthesia, but I would expect that there would be something in a non-surgical field. I imagine that surgical simulation could be quite different from non-surgical simulation.The authors pre-registered their study design, and this contributes to transparency in medical education research. However, there is an error in the web address provided; the study design can be found at clinicaltrials.gov.I found a few errors in Table 1. The wrong abbreviation is used once, and multiple values are marked as > even though the P value was non-significant. These should be listed as ≠.Finally, the authors concluded that the scores given by self, peer, and expert became more similar over time because the trainees had progressive training and increasing skill in the simulation. However, I wonder how much of the growing similarity in scores wasn't because after each performance, learners underwent standardized debriefings for 30 minutes, and this verbal assessment and feedback served as a type of training in how to use the rubric and assess their performance. The serial use of observation and standardized debriefings is similar to how raters are trained at some institutions.I think this paper is relevant to those who utilize self-assessment in their simulation program. It is well documented in the literature that learners are inaccurate in their self-assessments; this paper adds to the literature in specifying that as the same scenario is run repeatedly with debriefing, self-assessments become accurate.

Reviewer Expertise:

NA

No decision status is available

# Reviewer report: Reviewer response for version 1

**DOI:** 10.21956/mep.18892.r26978

**Published:** 2021-08-02

**Authors:** Augusto Scalabrini Neto

**Affiliations:** 1 University of Sao Paulo School of Medicine

This review has been migrated. The reviewer awarded 4 stars out of 5 Well structured paper, on a very important subject. Methodology is adequate. Population also adequate.Results are consistent and strong.Discussion and conclusions are relevant

Reviewer Expertise:

NA

No decision status is available
